# Supplementary material for: Reliability of COVID-19 data: An evaluation and reflection
Source: PLoS One. 2022 Nov 3;17(11):e0251470. doi: 10.1371/journal.pone.0251470 (PMC9632841; doi:10.1371/journal.pone.0251470)
Supplement: S1 File — (DOCX) [file pone.0251470.s001.docx]

## Washington state COVID-19 protocol

The first step in Washington state’s COVID-19 protocol involves identifying the symptomatic individual (S1 Fig). This individual will either be expressing symptoms of COVID-19, is a close contact of a case of COVID-19, is at-risk of transmitting COVID-19 due to various factors (below), or is a member of a group atypically prone to adverse COVID-19 outcomes undergoes testing through a healthcare provider or at a testing site. The provider/testing site collects information during the testing process, including contact information, demographic, race and ethnicity [[57]](https://www.zotero.org/google-docs/?0ONHNV)**.**

After identification, most tests are sent to clinical labs, and in certain situations, the specimen is sent to the Public Health Lab (which tends to have shorter wait times before returning a result). This is determined by various factors, most of which are used to identify people who work/live in environments where they are more likely to transmit the virus [[58, 59]](https://www.zotero.org/google-docs/?uLZthh). Next, regardless of which lab conducted testing, all results (positive, negative, and inconclusive) are shared with the local health jurisdiction (county health department). If the suspicion that an individual is carrying COVID-19 is high, a negative test will be repeated. Local health jurisdictions must be notified immediately of a positive result by telephone, regardless of the hour. Isolate or clinical specimens from a positive test must be sent to the Public Health Lab within two business days of the test result, if tested at a clinical lab [[43, 44, 57, 60]](https://www.zotero.org/google-docs/?RJiiRc).

After results are received, local health jurisdictions input case information into the Washington Disease Reporting System (WDRS) to report them to the Washington State Department of Health. The CDC is also notified through the WDRS. Data reported to the CDC is de-identified, and reported through HL7 messaging or a correctly formatted .CSV file. Case interviews/contact tracing are used to fill in any information not gathered during the initial test. Local health jurisdictions may not be given complete contact information for individuals, delaying notification [[45, 46, 61, 62]](https://www.zotero.org/google-docs/?iPs9wc).

Individuals are then notified of positive results in various ways, depending on where they had their test done. They may receive a call from the healthcare provider, from local health jurisdictions, or from their tester. Individuals are instructed to go into isolation. Contact tracers conduct interviews to determine how the virus was contracted, where possible. Contact tracers identify close contacts, who are notified, instructed to quarantine, and monitored for two weeks for symptoms of COVID-19. Monitoring of close contacts ceases after two weeks. Isolation for COVID patients ceases after their symptoms improve, they have had at least ten days pass since initial appearance of symptoms, and have gone three days without a fever. If never symptomatic, isolation ends ten days after a positive test [[45, 59, 63, 64]](https://www.zotero.org/google-docs/?uotaui). This process will have slight variations depending on the laws in a given state.

The CDC allows for several methods of reporting this data [[61]](https://www.zotero.org/google-docs/?tHzOy9). It is important to note that there may be numerous caveats of how COVID-19 data is reported and aggregated and ultimately making its way to the CDC. For example, on July 10, 2020 The U.S. Department of Health and Human Services (HHS) directed hospitals that perform COVID-19 testing or that use certain commercial labs to report using the HHS Protect System. Thus, some testing data bypassed the CDC and went directly to the HHS [[65]](https://www.zotero.org/google-docs/?L1SJHO).

**References**

[57. Interim COVID-19 Testing Guidance for Healthcare Providers. Wash State Dep Health. 2020;4.](https://www.zotero.org/google-docs/?rZywN7)

[58. Testing for COVID-19 :: Washington State Department of Health [Internet]. [cited 2021 Mar 16]. Available from: https://www.doh.wa.gov/Emergencies/COVID19/TestingforCOVID19](https://www.zotero.org/google-docs/?rZywN7).

[59. What is contact tracing? [Internet]. WASHINGTON STATE DEPARTMENT OF HEALTH; 2020. Available from: https://www.doh.wa.gov/Portals/1/Documents/1600/coronavirus/ContactTracingInfographic.pdf](https://www.zotero.org/google-docs/?rZywN7).

[60. WAC 246-101-201: [Internet]. [cited 2021 Mar 16]. Available from: https://app.leg.wa.gov/wac/default.aspx?cite=246-101-201](https://www.zotero.org/google-docs/?rZywN7).

[61. CDC. Labs [Internet]. Centers for Disease Control and Prevention. 2020 [cited 2021 Mar 16]. Available from: https://www.cdc.gov/coronavirus/2019-ncov/lab/reporting-lab-data.html](https://www.zotero.org/google-docs/?rZywN7).

[62. Letter to Lab Directors/Managers [Internet]. State of Washington Department of Health; 2020 [cited 2021 Mar 15]. Available from: https://www.doh.wa.gov/portals/1/documents/1600/coronavirus/20200307-covid-19labreporting.pdf](https://www.zotero.org/google-docs/?rZywN7).

[63. COVID-19 testing locations in King County - King County [Internet]. [cited 2021 Mar 16]. Available from: https://www.kingcounty.gov/depts/health/covid-19/testing.aspx](https://www.zotero.org/google-docs/?rZywN7).

[64. COVID-19 | Testing [Internet]. [cited 2021 Mar 16]. Available from: https://www.skagitcounty.net/Departments/HealthDiseases/coronavirusdriveup.htm](https://www.zotero.org/google-docs/?rZywN7).

[65. White House to hospitals: Bypass CDC, report COVID-19 data directly to HHS | Healthcare IT News [Internet]. [cited 2021 Apr 23]. Available from: https://www.healthcareitnews.com/news/white-house-hospitals-bypass-cdc-report-covid-19-data-directly-hhs](https://www.zotero.org/google-docs/?rZywN7).
