# Supplementary figures and images for: Reliability of COVID-19 data: An evaluation and reflection
Source: PLoS One. 2022 Nov 3;17(11):e0251470. doi: 10.1371/journal.pone.0251470 (PMC9632841; doi:10.1371/journal.pone.0251470)

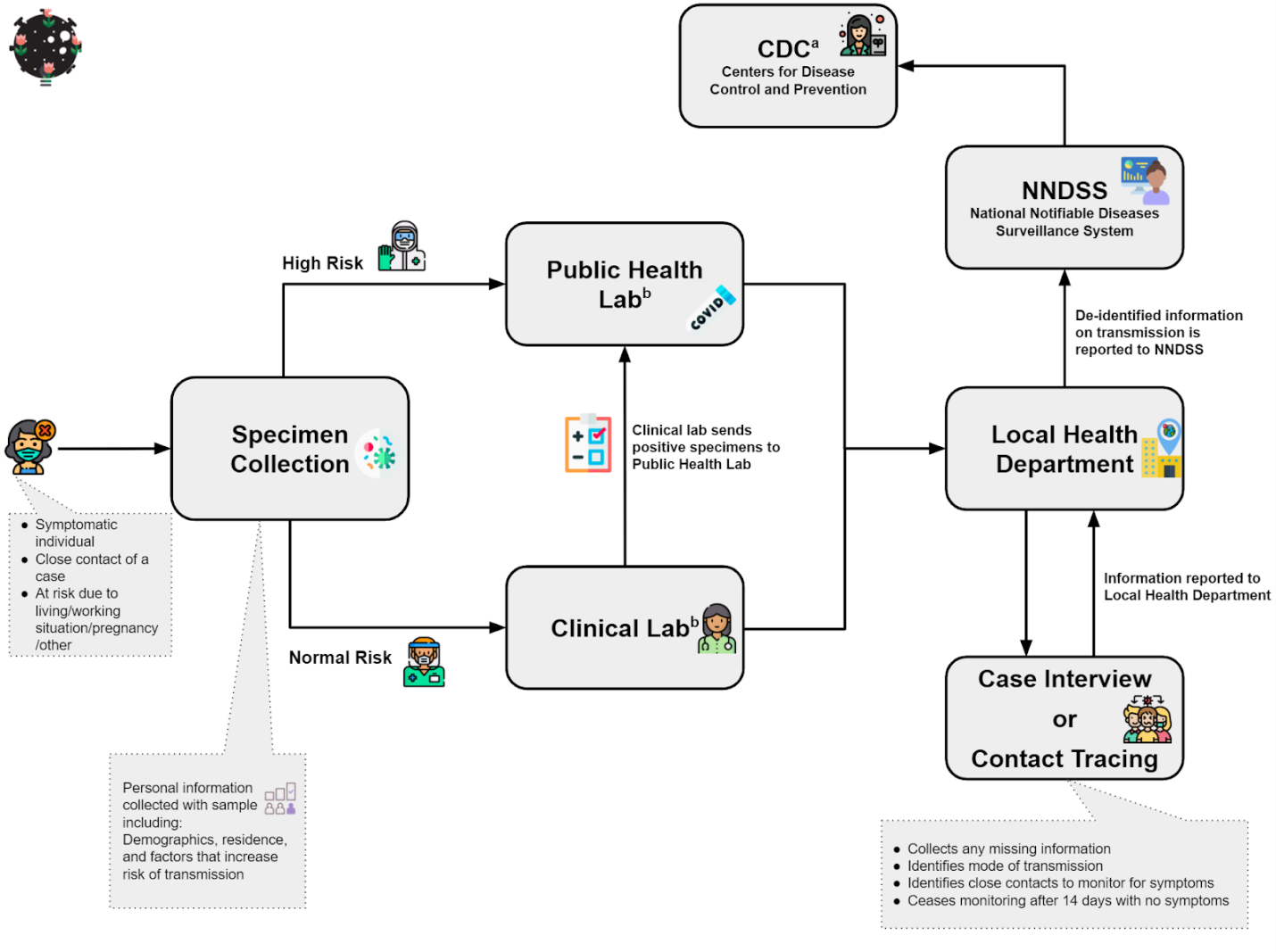

Supplement: S1 Fig — Note: Time period of this chart is July 10, 2020. Process may have been updated. (DOCX) [file pone.0251470.s002.docx]
