# Supplementary material for: Reliability of COVID-19 data: An evaluation and reflection
Source: PLoS One. 2022 Nov 3;17(11):e0251470. doi: 10.1371/journal.pone.0251470 (PMC9632841; doi:10.1371/journal.pone.0251470)
Supplement: Attachment — Submitted filename: Response to Reviewers.docx [file pone.0251470.s003.docx]

## Response to Editor

1. Thank you for stating the following in the Acknowledgments Section of your manuscript: "Thank you to BroadStreet and Franciscan Health for continued support."

We note that you have provided funding information that is not currently declared in your Funding Statement. However, funding information should not appear in the Acknowledgments section or other areas of your manuscript. We will only publish funding information present in the Funding Statement section of the online submission form. Please remove any funding-related text from the manuscript and let us know how you would like to update your Funding Statement. Currently, your Funding Statement reads as follows: "The author(s) received no specific funding for this work." Additionally, because some of your funding information pertains to [commercial funding//patents], we ask you to provide an updated Competing Interests statement, declaring all sources of commercial funding.

a. In your Competing Interests statement, please confirm that your commercial funding does not alter your adherence to PLOS ONE Editorial policies and criteria by including the following statement: "This does not alter our adherence to PLOS ONE policies on sharing data and materials.” as detailed online in our guide for authors http://journals.plos.org/plosone/s/competing-interests. If this statement is not true and your adherence to PLOS policies on sharing data and materials is altered, please explain how.
b. Please include the updated Competing Interests Statement and Funding Statement in your cover letter. We will change the online submission form on your behalf.
c. Please know it is PLOS ONE policy for corresponding authors to declare, on behalf of all authors, all potential competing interests for the purposes of transparency. PLOS defines a competing interest as anything that interferes with, or could reasonably be perceived as interfering with, the full and objective presentation, peer review, editorial decision-making, or publication of research or non-research articles submitted to one of the journals. Competing interests can be financial or non-financial, professional, or personal. Competing interests can arise in relationship to an organization or another person. Please follow this link to our website for more details on competing interests: http://journals.plos.org/plosone/s/competing-interests

*Response:* Thank you for these notes. We have now updated the ‘Acknowledgements’ section of the manuscript by removing any funding information. The funding statement and competing interests statement has also been updated in the cover letter.

2. Thank you for indicating that the map images in Figure 2 & 3 are not original. To ensure that they are compliant with our copyright policies, however, we ask that you provide additional information regarding the following points:
   a. Please indicate where the base maps/shapefiles used to create the map images were retrieved from, and provide a direct link to the source.
   b. Please indicate the source of the map data presented, and provide a direct link to the source, as relevant.
   c. Explain what software was used to create the map.
   d. Update the figure captions in your manuscript file accordingly.
   e. Remove the figure images from the manuscript file and upload them all instead as individual TIFF or PNG figure files, with the "Figure" item type in Editorial Manager.

*Response:* As previously stated, all figures within the manuscript are original. They were created by the authors using original data in open source R stats.
