# Supplementary material for: Reliability of COVID-19 data: An evaluation and reflection
Source: PLoS One. 2022 Nov 3;17(11):e0251470. doi: 10.1371/journal.pone.0251470 (PMC9632841; doi:10.1371/journal.pone.0251470)
Supplement: Attachment — Submitted filename: Response to Reviewers (3rd Revise-Resubmit).docx [file pone.0251470.s004.docx]

## Reviewer #1

The manuscript is satisfactory. Writing language is technically appropriate. Statistical analysis is appropriately reported.

1. Citations of references need to be rechecked. Citation of first reference not found in first paragraph of manuscript.

*Response:* The citation of the first reference is on the title page of the manuscript, as this is when the COVID-19 data project is first mentioned.

## Reviewer #4

The article is extremely interesting and relevant. The reviews carried out were adequate. Now, the manuscript technically sound, and do the data support the conclusions. The revision made was adequate and now we have a intelligible english. Also, The readability of the manuscript improved. The aim is now clear for me.

1. Was there any eligibility criteria for selection of volunteers as well as sources included in the study. If Yes; the criteria's can be listed?

*Response:* For both the selection of volunteers and sources included in the study there was selection criteria. Regarding sources used, these were chosen based on public availability of the datasets and collection methods, along with being cited and used by a variety of organizations such as the CDC and Google. This is stated on page 4 of the manuscript. A more detailed description of eligibility criteria for volunteers has now been included within the “Data Collection Methods” section of the manuscript on page 4. Volunteers were sourced from universities across the United States and were eligible to partake in this project if they had interest or experience in a public health-related field.

2. Finally there is no information regarding ethics approval. Considering that patient information, covid cases and deaths are being recorded, there should be information about the research ethics committee.

*Response:* This study only used publicly available data from media sources such as New York Times, USA Facts, and John Hopkins University. Publicly available data from county and state public health departments were also used. Seeing as aggregate data is used without providing any identifying information, the data is exempt from IRB approval. Furthermore, the authors are not affiliated with any academic institution. In short, because the data used was public, no ethics approval was sought or obtained.
